# Activation of *OsOxO2* by T-DNA Insertion Affects Plant Height and Leaf Angle in Rice

**DOI:** 10.1186/s12284-025-00879-0

**Published:** 2026-01-19

**Authors:** Xing-wen Hu, Hai-yan Wang, Xiao-lu Yu, David W. M. Leung, Yu-long Chen, Dan-hong Chen, Ke Chen, Xin-xiang Peng, E-e Liu

**Affiliations:** 1 College of Life Sciences, South China Agricultural University, Guangzhou, 510642 People’s Republic of China; 2 School of Biological Sciences, University of Canterbury, Private Bag 4800, Christchurch, 8140 New Zealand

**Keywords:** Auxin, Oxalate oxidase, OsIAAGLU, Plant architecture, T-DNA insertion

## Abstract

Inclination of leaf and plant height are important agronomic traits that are closely related to grain yields. Proteins involved in regulating free auxin (IAA) levels have a central role in modulating rice leaf angle and plant height. In this study, a rice mutant (named *lc*4) was found to have enlarged leaf angles and exhibit dwarfism compared to wild-type (WT) plants. Flanking analysis revealed that the mutation was caused by a T-DNA insertion at 270 bp upstream of the oxalate oxidase 2 (*OsOxO2*) ATG start codon. The insertion significantly increased the transcript levels of indole-3-acetic acid glucosyltransferase (*OsIAAGLU*), *OsOxO1*, *OsOxO2* and *OsOxO3* and OxO activity in *lc*4. Mutation of *OsIAAGLU* in *lc*4 led to partial restoration of the WT leaf angle and plant height phenotypes in *iaaglu*/*lc*4 which, however, still exhibited OxO activity comparable with that in *lc*4. On the other hand, mutation of *OsOxO2* in *lc*4 also resulted in restoration of the WT leaf angle and plant height phenotypes, accompanied by reduced OxO activity and transcripts of *OsIAAGLU*, *OsOxO1*, *OsOxO2* and *OsOxO3*. Nevertheless, overexpression of *OsIAAGLU*, but not *OsOxO2* in the WT background, resulted in the transgenic plants showing leaf angle and plant height similar to *lc*4. Overexpression of *OsOxO2* had no effect on the transcripts of *OsIAAGLU*, *OsOxO1* and *OsOxO3*. These results suggest that activation of *OsOxO2* and *OsIAAGLU* together resulted in altered plant architecture of *lc*4, while *OsIAAGLU* played a more direct role, *OsOxO2* might be involved in regulating *OsIAAGLU*, *OsOxO1* and *OsOxO3* in a chromosome position-dependent manner.

**Supplementary Information:**

The online version contains supplementary material available at 10.1186/s12284-025-00879-0.

## Introduction

Plant architectural attributes, including tiller number, leaf/tiller angle and plant height, are important agronomic traits in rice and play an important role in the relationships among photosynthetic efficiency, planting density and grain yield (Burgess et al. 2017; Huang et al. 2021b; Li et al. 2025; Weng et al. 2025). Many mutants affecting plant architecture have been identified genetically, genes underlying the mutant phenotypes are mainly related to auxin and/or brassinosteroid (BR) (Zhang et al. 2009, 2015; Du et al. 2012; Zhao et al. 2013; Yamamuro et al. 2000; Sakamoto et al. 2006). Auxin plays vital roles in maintaining normal development of rice blade because it is involved in the elongation of parenchyma cells in the lamina joint connecting the leaf and the sheath and regulation of leaf angle (Zhao et al. 2013; Zhang et al. 2015). Therefore, reduction in free auxin content in lamina joint tissues resulted in increased flag leaf angle (Zhang et al. 2009, 2015; Du et al. 2012; Zhao et al. 2013). A number of mutants with altered IAA levels and responses to IAA also showed modified leaf angle. IAA early responsive genes, including Aux/IAA, IAA receptor TIR1, IAA response factors ARF and GH3, have been reported involved in regulating leaf angle in rice. Transgenic plants overexpressing *OsIAA1* (a member of rice Aux/IAA family) displayed dwarfism and enlarged leaf angle phenotypes (Song et al. 2009). *OsTIR1*- or *OsAFB2*-RNAi transgenic lines also showed enlarged leaf inclination (Bian et al. 2012). Rice gain-of-functions mutant *tld1*-*D* (*OsGH3-13*) showed enlarged leaf angle, dwarfism and tiller number increase phenotypes (Zhang et al. 2009). Similarly, typical IAA-deficient phenotypes including dwarfism and enlarged leaf angle were also observed in plants overexpressing *OsGH3-1* (Zhao et al. 2013) or *OsGH3-2* (Du et al. 2012). Additionally, overexpression of *ZmIAAGLU* in Arabidopsis made the plants insensitive to IAA, and to display shorter roots and curly leaf blade (Ludwig-Müller et al. 2005). This gene encodes an enzyme that catalyzes IAA conjugation with uridine diphosphate glucose (UDPG) to form IAA-glucose (Szerszen et al. 1994). OsIAAGLU which shares 67% identity with ZmIAAGLU can also catalyze the reaction of free IAA with glucose to generate IAA-glucose. Transgenic rice plants overexpressing *OsIAAGLU* exhibited dwarfism, increased leaf angle and tiller number, and shorter spike (Choi et al. 2012; Yu et al. 2019). These findings demonstrate that endogenous free IAA levels play a crucial role in regulating rice leaf angle and plant height. Despite the importance of IAA in leaf angle regulation has been well established, the precise mechanisms underlying its fine-tuning remain unclear.

Besides auxin, BR, ethylene, GA and other hormones could also participate synergistically in regulation of rice plant architecture (Shimada et al. 2006; Sakamoto et al. 2006; Hong et al. 2002, 2003; Yamamuro et al. 2000). Additionally, *ILA1* encodes a Ser/Thr kinase which can regulate leaf angle through altering strength of mechanical tissue and cell wall composition in lamina joint. Leaf angle of *Ila1*-deficent mutant increased remarkably, but the sensitivity to BR was similar to WT (Ning et al. 2011). Mutation of *LC2* encoding a vernalization insensitive 3-like protein resulted in enlarged leaf angle due to increased cell division in the adaxial region of the lamina joint (Zhao et al. 2010). LPA1, a typical Cys-2/His-2 zinc finger protein, can also regulate leaf angle by modulating the cell elongation in the adaxial epidermis of lamina joint (Wu et al. 2013). Therefore, the regulation of leaf angle is very complex involving a multitude of factors, the molecular mechanism remains to be elucidated.

Although mutants associated with plant architecture are numerous, most genes underlying the mutant phenotype have been found to act through altering cell division or composition of cell wall (Ueguchi-Tanaka et al. 2000; Shimada et al. 2006; Tong et al. 2014; Liu et al. 2024). Oxalate oxidase (OxO), as a crucial members of the germin-like proteins which perform a variety of biological functions in plant development, stress response, and pathogen resistance in crops (Manosalva et al. 2009), catalyzes conversion of oxalate into H_2_O_2_ and CO_2_, simultaneously accompanying with the release of Ca^2+^ (Lane et al. 1993). It has been considered to be involved in the modification of cell wall architecture, because OxO is localized in cell wall and within the cell type which characteristically restricted in the extent of cellular enlargement, such as coleorhiza, coleoptile, scutellum and vascular bundles (Lane et al. 1993; Caliskan and Cuming 1998), but their definite physiological functions are still unknown.

In rice genome, four tandemly duplicated OxO-encoding genes (*OsOxO1*, *OsOxO2*, *OsOxO3*, and *OsOxO4*) are clustered on chromosome 3, exhibiting over 90% sequence similarity at both nucleotide and amino acid levels. These four genes have different temporal and spatial patterns of expression (Carrillo et al. 2009). Here, we identified a rice oxalate oxidase 2 (*OsOxO2*) T-DNA mutant, which exhibited enlarged leaf angles and dwarfism compared to the wild-type plants (WT). T-DNA insertion elevated the transcript levels of *OsOxO1*, *OsOxO2*, *OsOxO3* and *OsIAAGLU*. Therefore, this T-DNA mutant was of particular interest to study regulation of phenotypes via in situ gene activation. Studies here on the mechanisms of how the T-DNA insertion results in the altered plant architecture support the hypothesis that OsOxO2 is involved in IAA signaling regulation of plant architecture by modulating the in situ expression level of *OsIAAGLU* in rice.

## Materials and Methods

### Plant Materials and Growth Conditions

Rice (*Oryza sativa* L) cv. Dongjin (DJ) was used to generate *OsOxO2*/*OsIAAGLU* overexpression transgenic plants. The *lc*4 mutant of rice was identified from the RiceGE database (http://signal.salk.edu/cgi-bin/RiceGE). After germination, seedlings were grown in Kimura B complete nutrient solution (Yoshida et al. 1976) under experimental conditions as described in the respective figure legend. For maturation-stage phenotyping, plants were grown in a paddy field in Guangzhou.

### Identification of the *lc*4 Mutant

The insertion site of T-DNA was determined by PCR amplification using T-DNA-specific primer LB (matching the left border of the inserted T-DNA pGA2715) and gene-specific primer RP (matching the flanking sequence of the expected insertion site in the downstream of *OsOxO2* gene), followed by sequencing to identify the insertion site. Homozygous T-DNA insertion mutants (with both alleles disrupted) were identified by PCR-based methods with gene-specific primer pairs LP (matching the flanking sequence of the expected insertion site in the upstream of *OsOxO2* gene) and RP together with RP and LB (Jeong et al. 2002). The single T-DNA insertion event in *lc*4 was verified by analyzing the segregation ratio in progeny from heterozygous mutants (one allele disrupted).

### Expression Analysis of *OsOxO1-4* and *OsIAAGLU*

Approximately 0.1 g fresh rice leaves were collected, then frozen in liquid nitrogen immediately and ground into a fine powder. Subsequently, total RNA was extracted with RNAiso Plus (Takara) and reverse-transcribed following the manufacturer’s instructions (Vazyme Biotech, China). The transcripts of *OsOxO1-4* were analyzed by semi-quantitative RT-PCR, while the expression of *OsIAAGLU* was quantified by qRT-PCR on PTC200 (BIO-RAD) using SYBR green master mix (Bimake, China). *Actin* was used as an internal control. The level of *OsIAAGLU* transcript was calculated using the following formula: Target gene transcript = 2^−(Ct Target gene−Ct Actin)^. All primer sequences are listed in Table S1.

### Construction of Rice Transgenic Plants

For the construction of *OsIAAGLU*OE and *OsOxO2*OE overexpression vector, the full-length *OsIAAGLU*/*OsOxO2* coding sequence was amplified using RT-PCR and cloned into the poX vector harboring the Ubi promoter (provided by Professor Yao-Guang Liu, College of Life Sciences, South China Agricultural University, China). The vectors were subsequently introduced into *Agrobacterium tumefaciens* strain EHA105 for transformation of the calli of rice variety Dongjin (DJ). *iaaglu*/*lc*4 or *oxo2*/*lc*4 mutants were constructed using the CRISPR/Cas9 system following the protocol described by Ma et al. (2015). First, one gRNA specific to *OsOxO2*/*OsIAAGLU* was inserted into the vector pYLCRISPR/Cas9Pubi-B (glyphosate resistance in rice), then the vector was introduced into *Agrobacterium tumefaciens* strain EHA105 for transformation of *lc*4 calli using the method of Hiei et al. (1994). The mutation of *OsOxO2*/*OsIAAGLU* in transgenic plants was genotyped by PCR amplification of the target region using DNA extracted from their leaves, followed by sequencing to verify mutations. The primer sequences used for vector construction and amplification of the target region are listed in Table S1.

### Assay of Oxalate Oxidase (OxO) Activity

OxO activity was carried out according to Zhang et al. (1996). Fresh tissue (about 5 mg) was homogenized using liquid nitrogen and the powder used for determination of OxO activity. The assay mixture contained the powder, 40 mmol/L succinic acid/NaOH buffer, pH 3.8, 60% ethanol (V/V), 0.4 mmol/L oxalic acid, 0.025% N, N-dimethylaniline, 0.1 mg/mL 4-aminoantipyrine and 5 U/mL of horseradish peroxidase. The mixture was incubated at room temperature for 6 min and trichloroacetic acid (0.1%) was added to terminate the reaction, then the absorbance of the supernatant was measured at 555 nm after the mixture were centrifuged at 12,000 g for 3 min at 4℃. OxO activity was determined as the amount of H_2_O_2_ (µM) produced in 1 g tissue per min. For in-gel OxO activity staining, proteins were extracted from leaves with 50 mmol/L Tris-HCl buffer (pH = 7.5) and separated in 7.5% native-PAGE. After gel electrophoresis, the gel was immersed in a staining solution containing 5 U/mL peroxidase (POD), 2 mmol/L oxalate, 0.5 mg/mL 4-chloro-1-naphthol, 60% ethanol (V/V) and 40 mmol/L succinic acid/NaOH buffer (pH 3.8) at room temperature until visible bands of enzyme activity could be observed.

### Protein Immunoblotting

The proteins were extracted from the leaves of rice seedlings using 50 mmol/L Tris-HCl buffer (pH 7.5). The extracts were mixed with 2×SDS loading buffer (100 mmol/L pH = 6.8 Tris-HCl buffer containing 20% glycerol, 4% SDS, 4% mercaptoethanol and 0.04% bromophenol blue) before the proteins in the extracts were separated using 12.5% SDS-PAGE. After electrophoresis, the proteins were then transferred to nitrocellulose / PVDF membranes. Anti-His-OsOxO4 prepared in our laboratory was used as the primary antibody at 1: 2000 dilution. Horseradish peroxidase (HRP)-conjugated anti-rabbit (BBI D110011) was used as the secondary antibody. Immunodetection was performed using an ECL Enhanced Plus Kit (ABclonal, Wuhan, China) and detected using a ChemiScope 6000 Touch chemiluminescence imaging system (Clinx).

## Results

### A pGA2715 T-DNA Vector Tagged 270 bp Upstream of *OsOxO2* Resulted in Enhanced Levels of *OsOxO1*, *OsOxO2*, *OsOxO3* and *OsIAAGLU* Transcripts

**Fig. 1:**
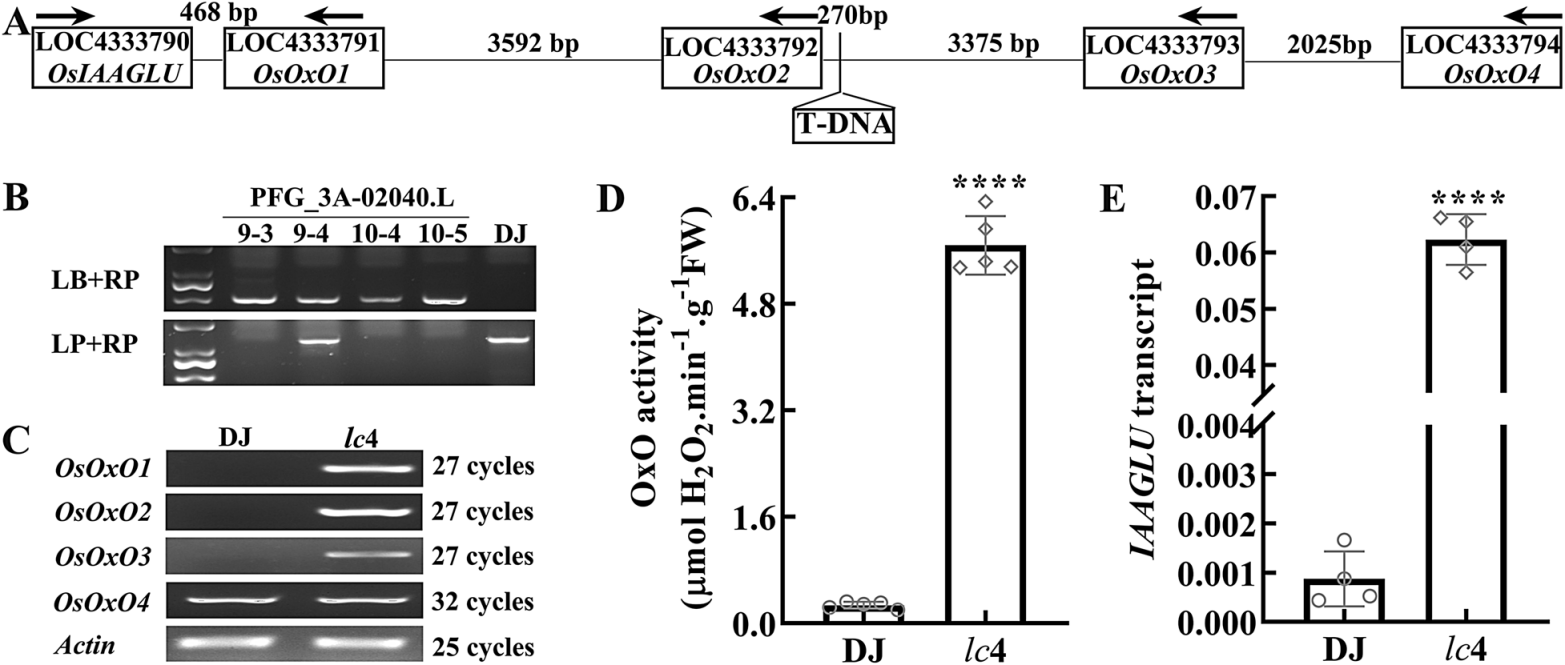
T-DNA insertion in *OsOxO2* increased OxO activity and *OsIAAGLU* transcript levels. (**A**) schematic diagram of the T-DNA insertion site in PFG_3A-02040.L and the genes in its flanking genomic region, arrowheads indicate the transcriptional direction of each gene. (**B**) confirmation of the T-DNA insertion in PFG_3A-02040.L and identification of the homozygous lines by PCR amplification using genomic DNA and primers sets (LB+RP and LP+RP). (**C**) semi-quantitative RT-PCR analysis of *OsOxO1-4* transcription levels in the leaves of wild-type (DJ) and *lc*4 seedlings. (**D**) OxO activity in DJ and *lc*4, data represent as means ± SD (n=5). (**E**) relative *OsIAAGLU* transcript levels in DJ and *lc*4, calculated as described in Materials and Methods, data are shown as mean ± SD (n = 4). The means±SD assigned with asterisks indicate significant differences between mean values by Student’s *t*-test (*P*<0.0001)

To elucidate the physiological functions of oxalate oxidase (OxO) in rice, a T-DNA (Transfer DNA) insertion line (PFG_3A-02040.L) associated with the Os03g0693800 locus (*OsOxO2*) obtained from the Rice Functional Genomic Express Database (http://signal.salk.edu/cgi-bin/RiceGE) was screened. To determine the T-DNA insertion site in PFG_3A-02040.L, PCR amplification and sequencing were performed with LB (primer specific to the left border of the inserted T-DNA pGA2715) and RP (downstream primer specific to the flanking sequence of the expected insertion site in the native *OsOxO2* gene) as primers, which revealed that the T-DNA was inserted 270 nucleotides upstream of the *OsOxO2* ATG start codon (Figure S1 and Fig. 1A). PCR amplification using rice genomic DNA as the template and primers sets LB + RP, together with LP (upstream primer specific to the flanking sequence of the expected insertion site in the native *OsOxO2* gene) + RP showed the progenies of PFG_3A-02040.L 9 − 3, 10 − 4 and 10 − 5 appeared to be homozygous mutants (Fig. 1B), because amplicon only with the LB + LP combination was observed and no amplicon with the LP + RP combination in the above lines. Subsequently, the 10 − 4 mutant, named herewith as *lc*4, was used in following experiments. Compared to WT, the transcript levels of *OsOxO1*, *OsOxO2* and *OsOxO3* (Fig. 1C) were significantly elevated in the leaves of *lc*4, OxO activity also increased remarkablely in *lc*4 mutants and represented 20-fold increase compared to WT controls (Fig. 1D). However, OxO activity gel staining in native PAGE and immunoblot assay (Figure S2) showed OsOxO2 is the predominant OxO isoform present in *lc*4 mutant leaves, because only one band was present in *lc*4 and the electrophoretic mobility of the band was similar to that of in *OsOxO2*OE/DJ. Additionally, the expression of *OsIAAGLU*, a gene encoding an IAA-conjugating enzyme that generates IAA-glucose by conjugating free IAA to glucose (Yu et al. 2019) was also upregulated in *lc*4 (Figs. 1A and E).

### The Activation of *OsOxO2* in Rice Resulted in Increased Leaf Angle and Semi-dwarfism Phenotype

**Fig. 2:**
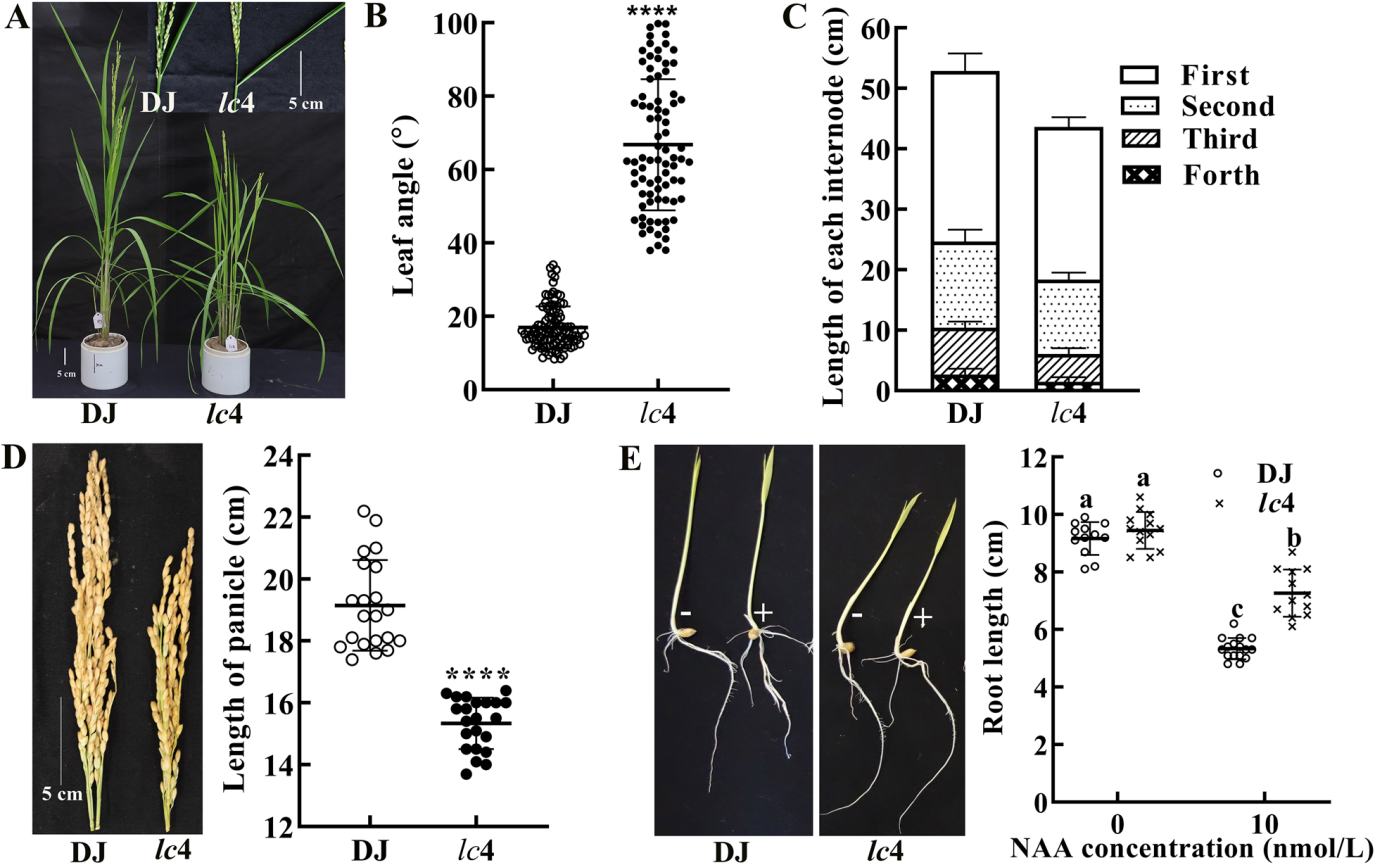
Characterization of the phenotype of the *lc*4 T-DNA insertion mutant of rice. (**A**) the phenotype and flag leaf of representative wild-type (DJ) and the *lc*4 mutant plants. (**B**) quantification of leaf angle of plants depicted in (**A**). (**C**) the length of the internodes (n≥18), and panicle length (**D**) of DJ and *lc*4 plants (n=21) at 120 days from sowing. (**E**) the phenotype and root length of DJ and *lc*4 seedlings treated with Kimura B complete nutrient solution containing NAA kept in the dark for 5 d. “-” and “+” represent 0 and 10 nmol/L NAA. The means±SD assigned with asterisks indicate significant differences between mean values by Student’s *t*-test (*P*<0.0001). The means±SD of root length assigned with different letters were significantly different as determined by one-way analysis of variance (ANOVA) with Duncan test (*P* <0.05)

Compared with WT rice plants, *lc*4 showed increased leaf angle and decreased plant height at 4-leaf-stage (Figure S3A-C) and the differences were more obvious at the booting stage (Fig. 2A-C). Moreover, the flag leaf angle and adaxial distance in the lamina joint of *lc*4 were increased, while the abaxial distance in the lamina joint was reduced (Figure S3D-E). However, the culm of *lc*4 was shorter than that of WT plants and the length of each internode was uniformly reduced in *lc*4 plants (Fig. 2C). Additionally, the length of panicle was also decreased compared to WT (Fig. 2D). Because the expression of *OsIAAGLU*, encoding an IAA-conjugating enzyme, was increased in the leaves of *lc*4, the sensitivity to NAA in *lc*4 mutant was investigated in a root elongation assay, the results showed the growth inhibition induced by exogenous NAA in roots of *lc*4 seedlings was decreased in comparison with WT plants (Fig. 2E), suggesting that modified plant architecture of *lc*4 might related to IAA. To explore that the increased leaf inclination and semi-dwarf phenotype of *lc*4 was due to the T-DNA insertion, the co-segregation analysis was carried out by analyzing the phenotype of progeny from heterozygous PFG_3A-02040.L mutant 3A9-2. Plants with T-DNA insertion and high level of OxO activity all exhibited increased leaf inclination and semi-dwarf phenotype, whereas those without insertion resembled WT (Figure S4). These results indicated that the T-DNA insertion was linked to enhanced OxO activity as well as alterations in plant architecture.

### Transgenic Rice Plants Overexpressing *OsIAAGLU*, but not *OsOxO2*, Displayed Phenotype Resembling *lc*4

**Fig. 3:**
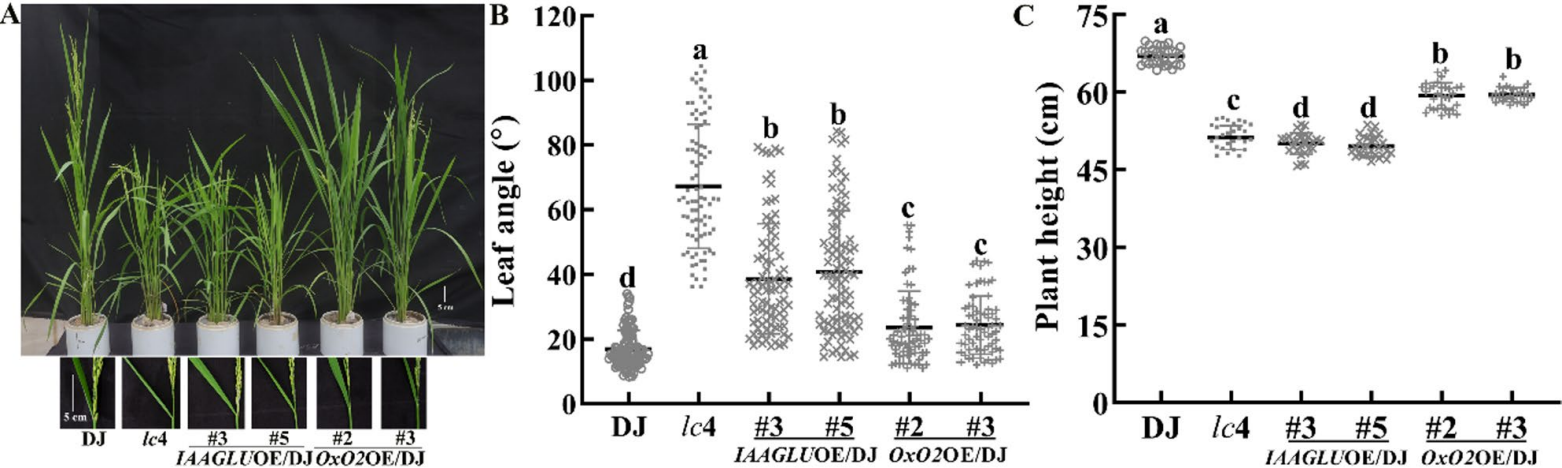
Transgenic rice plants overexpressing *OsIAAGLU*, not *OsOxO2*, in the wild-type background of rice variety Dongjin (DJ) displayed phenotype similar to *lc*4. (**A**) the phenotype of representative plants of DJ, the *lc*4 mutant, those overexpressing *OsIAAGLU* (lines #3 and #5) or *OsOxO2* (lines #2 and #3) and flag leaf at the heading stage. (**B**) and (**C**) quantification of leaf angle at the filling stage and plant height at the maturation stage. The means ± SD of leaf angle and plant height assigned with different letters were significantly different as determined by one-way analysis of variance (ANOVA) with Duncan test (*P* < 0.05)

The insertion of T-DNA caused upregulated expression of both *OsOxO1*-3 and *OsIAAGLU* (Fig. 1C and E). Since the T-DNA was inserted into the promoter of *OsOxO2* while *OsIAAGLU* has been shown to significantly regulate plant architecture (Yu et al. 2019), we focused on investigating whether *OsIAAGLU* and *OsOxO2* would contribute to the alterations of plant architecture in *lc*4. For this purpose, we examined the architecture in plants overexpressing *OsIAAGLU* or *OsOxO2*. Overexpression of *OsIAAGLU* (driven by a constitutive promoter, pUbi: *OsIAAGLU*) in WT of the DJ background resulted in significantly exaggerated leaf angles (Fig. 3A and B) and decreased plant height (Fig. 3A and C) compared to WT. However, overexpression of *OsOxO2* in WT of the DJ background, unlike its increased expression in *lc*4, only slightly increased leaf angle and decreased plant height compared to WT (Fig. 3), although there was a remarkable increase in OxO activity in the leaves (Figure S2). These results suggest that overexpressing *OsIAAGLU*, but not those of *OsOxO2*, resulted in enhanced leaf angle and dwarfism, suggesting that the increased leaf angle and dwarfism phenotypes of *lc*4 are mainly related to an increase in *OsIAAGLU* expression in *lc*4, rather than the elevated expression levels of *OsOxO2*.

### Partial Restoration of WT Phenotype by Mutation of *OsIAAGLU* in *lc*4

**Fig. 4:**
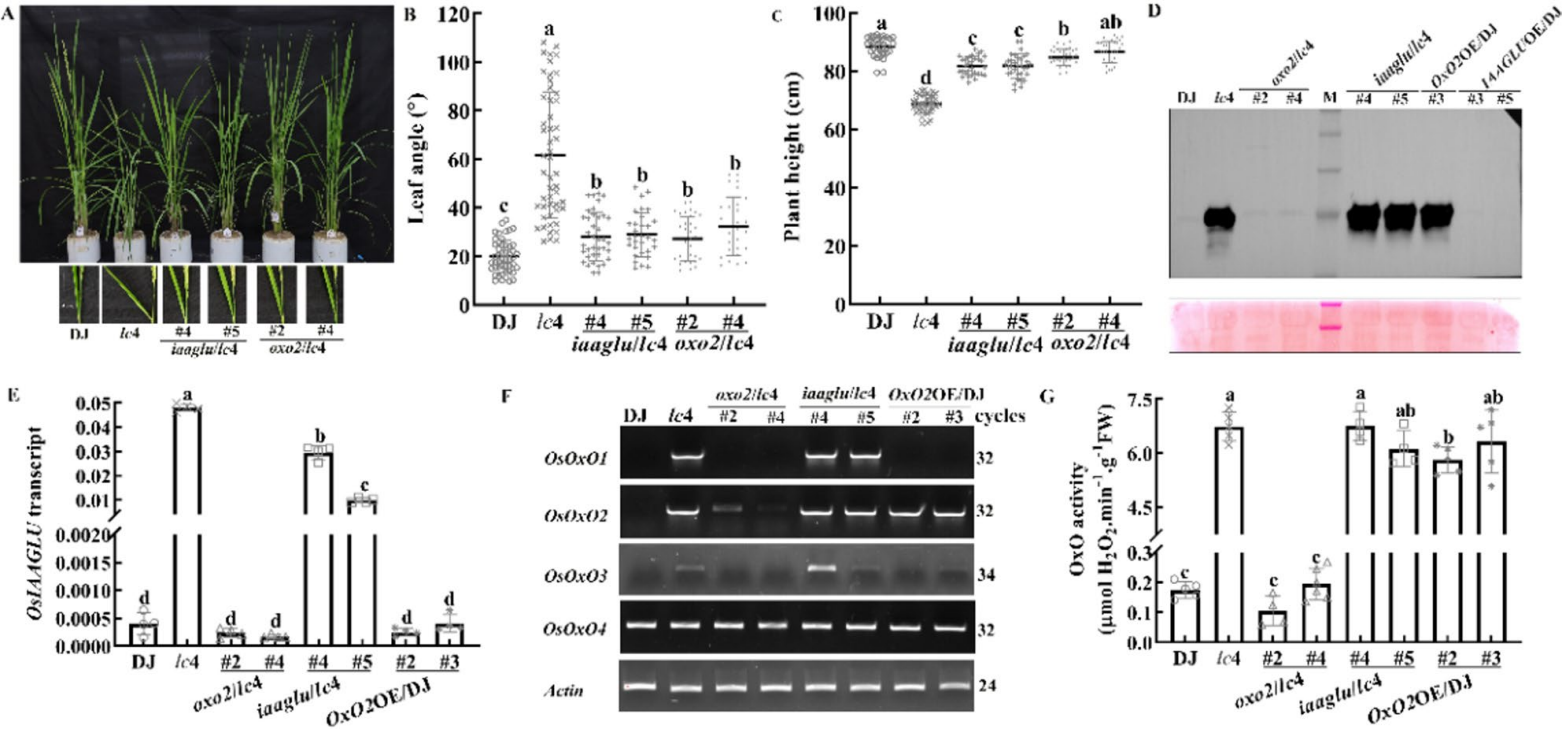
Mutation of *OsIAAGLU* or *OsOxO2* in the *lc*4 background led to restoration of wild-type (WT) phenotype. (**A**) phenotype at the heading stage and leaf angle at the filling stage of representative plants of WT (DJ), the *lc*4 mutant, *iaaglu*/*lc*4 (lines #4 and #5), and *oxo2/lc*4 (lines #2 and #4). (**B**) quantification of leaf angle of plants depicted in (**A**). (**C**) plant height at the maturation stage. (**D**) immunoblot analysis of OxO protein levels in the leaves (second leaves from top to bottom) of 5-leaf stage wild type (DJ), the *lc*4 mutant, *oxo2*/*lc*4 (lines #2 and #4), *iaaglu*/*lc*4 (lines #4 and #5), *O*s*OxO2*OE/DJ (lines #3) and *IAAGLU*OE/*lc*4 (lines #3 and #5). Ponceau-stained ribulose-1, 5-bisphosphate carboxylase/oxygenase large subunit (RbcL) is shown as a loading control. (**E**) the expression levels of *OsIAAGLU* in WT, *lc*4, *oxo2*/*lc*4 (#2 and #4), *iaaglu*/*lc*4 (#4 and #5) and *OsOxO2*OE/DJ (#2 and #3) grown under natural sunlight to the 4-leaf stage, quantitative data was calculated using the formula in Materials and Methods and are presented as mean ± SD (*n* = 4). (**F**) the expression levels of *OsOxO1*, *OsOxO2*, *OsOxO3*, and *OsOxO4* in the same genotypes as in (**E**). (**G**) OxO activity in the same genotypes as in (**E**), quantitative data of OxO activity are shown as means ± SD (*n* ≥ 4). The means ± SD of leaf angle, plant height, *OsIAAGLU* transcripts and OxO activity assigned with different letters were significantly different as determined by one-way analysis of variance (ANOVA) with Duncan test (*P* < 0.05)

To investigate whether the increased expression of *OsIAAGLU* in *lc*4 is directly associated with the altered plant architecture in *lc*4, we mutated *OsIAAGLU* in *lc*4 by the CRISPR-Cas9 system (Figure S6A). The leaf inclination as well as plant height in the transgenic plants were significantly different from those of *lc*4 and appeared to be partially restored back to the WT phenotype (Fig. 4), which confirms that *OsIAAGLU* plays a major role in affecting the plant height and leaf angle phenotype of *lc*4. Although transgenic plants with different mutations in *OsIAAGLU* of *lc*4 (*iaaglu*/*lc*4) had reduced levels of *OsIAAGLU* transcripts in leaves compared to *lc*4, the levels were still significantly higher than that of WT (Fig. 4E). This indicates that the transcription of *OsIAAGLU* is influenced by other factors. To further confirm the role of *OsIAAGLU* in regulating the *lc*4 phenotype, we examined OxO protein, *OsOxOs* transcripts and OxO activity in *iaaglu*/*lc*4. The results showed that OxO protein, *OsOxOs* transcripts and OxO activity in the leaves of *iaaglu*/*lc*4 together with plant height at 3-leaf seedlings stage were not significantly different from those in *lc*4 (Fig. 4D, F, G and Figure S5A). In addition, the OxO protein levels remained unchanged in *IAAGLU*OE/DJ (Fig. 4D). All the above data not only suggest that the expression level of *OsIAAGLU* does not influence OxO activity and the transcripts of *OsOxO1*-*4* which are co-localized on the same chromosome, but also further confirm that the *lc*4 phenotype is directly regulated by the expression of *OsIAAGLU* rather than that of *OsOxOs*.

### Restoration of WT Phenotypes by Mutation of *OsOxO2* in *lc*4

Besides mutation of *OsIAAGLU* in the *lc*4 background partially restored WT phenotypes, these plants maintained comparable OxO activity along with transcript levels of *OsOxO1*, *OsOxO2*,* OsOxO3* and *OsIAAGLU* with *lc*4. Therefore, *OsOxO2* was mutated in *lc*4 by the CRISPR-Cas9 system (Figure S6A). Compared to WT plants, the leaf angle and plant height in *oxo2*/*lc*4 were mostly restored (Fig. 4B and C). Additionally, compared to *lc*4, in the leaves of *oxo2*/*lc*4, not only was *OsOxO2* transcript significantly reduced, but OxO activity, OxO protein, and the transcripts of *OsOxO1*, *OsOxO3* and *OsIAAGLU* were also significantly decreased to the levels similar to those in WT (Fig. 4D-G), while T-DNA were present in the original position (Figure S6B). These results suggest that *OsOxO2* may regulate the *OsOxO1*, *OsOxO3* and *OsIAAGLU* expression, thereby modulating rice plant height and leaf inclination. However, in the leaves of *OsOxO2*OE/DJ, no significant changes were detected in the transcripts of *OsIAAGLU*, *OsOxO1* and *OsOxO3* (Fig. 4E and F), indicating that ectopic expression of *OsOxO2* does not affect the transcripts of *OsIAAGLU*, *OsOxO1* and *OsOxO3*. Based on the above results, we propose that the regulatory effect of *OsOxO2* may be related to its chromosomal position relative to these three genes. Ectopic expression of *OsOxO2* in WT had no effect on transcripts of *OsIAAGLU*, *OsOxO1* and *OsOxO3*, but significantly increased OxO protein levels and OxO enzymatic activity in leaves (Fig. 4D and G), indicating that *OsOxO2*-encoded protein possesses OxO activity.

## Discussion

### The Increase in *OsIAAGLU* Transcript Level Plays a Predominant Role in Alterations of Plant Architecture in *lc*4

Rice genome presents four tandem repeat *OxO* genes (LOC4333791-LOC4333794 and herewith referred to as *OsOxO1*-*4*) on chromosome 3 coding for OxO. Similar sequence and the very close genomic location for *OsOxO1-4* suggest their functional redundancy and duplication. Here, we identified a *OxO2* T-DNA mutant with significantly increased OxO activity and alternations in plant architecture. Compared to wild-type (WT) plants, leaf angle of *lc*4 increased at the 4-leaf-stage and more significantly at the booting time. Similarly, the differences in plant height between WT and *lc*4 became more obvious at the booting time than at the seedling stage. Moreover, the phenotype of enlarged leaf angle and semi-dwarfism in the co-segregated progeny that also had higher OxO activity and T-DNA insertion than the progeny from the heterozygous PFG_3A-02040.L mutant 3A9-2. Because cell wall OxO might be involved in the development from an immature cell wall to a more rigid, mature cell wall by promoting peroxidase activity in the presence of a supply of hydrogen peroxide (Wakabayashi et al. 2011). OxO is localized within cell type which is characteristically restricted in the extent of cellular enlargement. For example, during wheat seed germination, OxO is found in tissues such as coleorhiza, coleoptile, scutellum and vascular bundles. This is consistent with the hypothesis that the biological function of OxO is to restrict cell growth by participating in cell-wall restructuring through the localized H_2_O_2_ provision for cross–linking of wall components (Caliskan and Cuming 1998). Auxin response factors (ARFs), OsARF6 and OsARF17 are highly expressed in lamina joint tissues. The *osarf*6 and *osarf*17 mutants displayed an exaggerated flag leaf angle due to the reduced secondary cell wall deposition of the lamina joint sclerenchymatous cells (Huang et al. 2021a). Asymmetric cell division and elongation as well as cell wall development on the adaxial or abaxial sides of lamina joint can bring about leaf angle changes (Xu et al. 2021). Therefore, aberrant plant architectural phenotype of *lc*4 was originally thought to be due to an increase in OxO expression. In the transgenic plants overexpressing *OsOxO2* in the DJ (WT) background, the levels of *OsOxO2* transcript and OxO activity were increased greatly, but their phenotype was only slightly similar to *lc*4. In the transgenic plants of the DJ background overexpressing *OsIAAGLU*, which is in close proximity to *OsOxO1* on the chromosome, the increased level of *OsIAAGLU* transcript was also accompanied with phenotypes including the decreased plant height, sensitivity to NAA and enlarged leaf inclination similar to *lc*4. Likewise, decreased plant height and panicle length in transgenic rice overexpressing *OsIAAGLU* were also reported by Choi et al. (2012), while decreased plant height and increased leaf angle in rice overexpressing *OsIAAGLU* by reducing IAA content was reported by Yu et al. (2019). Conversely, with mutation of *OsIAAGLU* in *lc*4, the phenotype of the *iaaglu*/*lc*4 resembled WT, although there were still significant differences between WT and *iaaglu*/*lc*4 as far as plant height and leaf angle were concerned. In addition, *iaaglu*/*lc*4 had comparable levels of OxO activity as well as *OsOxO1* and *OsOxO2* transcripts with *lc*4, suggesting *OsIAAGLU* might play a predominant role in alteration of *lc*4 phenotypes. Strikingly, the phenotypes including leaf angle and plant height of the plants (*oxo2*/*lc*4) with mutation of *OsOxO2* in the *lc*4 background were restored close to those of DJ. In addition, the levels of *OsIAAGLU*, *OsOxO1* transcript and OxO activity in the leaves of *oxo2*/*lc*4 were comparable with those in DJ, suggesting that OsOxO2 also plays an important role in the regulation of plant height and leaf angle. Taken together, the enhanced expression of *OsIAAGLU* and *OsOxO2* in *lc*4 resulted in altered plant architecture. It appeared that *OsIAAGLU* could play a direct role in the regulation of plant architecture, and a regulatory relationship might exist between *OsOxO2* and *OsIAAGLU*.

### *OsOxO2* Might Be Involved in Regulating *OsIAAGLU*, *OsOxO1* and *OsOxO3* in a Chromosome position-dependent Manner

Activation tagging is a method to generate dominant mutations in plants or plant cells by random insertion of a T–DNA carrying constitutive enhancer element, which can cause transcriptional activation of flanking plant genes (Memelink 2003; Jeong et al. 2002). Rice nicotianamine synthase gene (*OsNAS3*) transcript levels were 60- and 30-fold higher in the leaves of seedlings from 2 independent activation-tagged alleles *OsNAS3*-D1 (35 S enhancer elements were inserted approximately 1.5 kb downstream of an ORF of *OsNAS3*) and *OsNAS3*-D2 (the enhancer elements were inserted approximately 1.9 kb downstream of the *OsNAS3* ORF), respectively, compared with WT (Lee et al. 2009). In this study, a T-DNA, i.e. pGA2715, was inserted at 270 bp upstream of *OsOxO2* CDS. Therefore, the enhanced OxO activity and expression of *OsOxO2* in the *lc*4 mutants might be related to the 4 copies of 35 S enhancers which were inserted next to the left border of T-DNA pGA2715. Likewise, 4 of the 10 candidate genes harboring pGA2715 and the enhancers within 4.5 kb displayed elevated expression (Jeong et al. 2002). However, transcript levels of *OsOxO1* and *OsOxO3* which are neighboring genes of *OsOxO2* were also increased significantly in the *lc*4 mutants. In addition, *OsIAAGLU* with its 3′ UTR being 468 bp away from the 3′ UTR of *OsOxO1* also showed enhanced expression in *lc*4, which seemed to play a predominant role in the altered plant architecture. Strikingly, mutation of *OsOxO2* in *lc*4 reduced the expression of *OsIAAGLU*, *OsOxO1* and *OsOxO3* as well as OxO activity in the leaves of *oxo2*/*lc*4 to the same level as those in DJ, suggesting a regulatory relationship might exist between *OsOxO2* and *OsOxO1*, *OsOxO3*, *OsIAAGLU*. However, the transcripts of *OsOxO1*, *OsOxO3* and *OsIAAGLU* are not up-regulated in the leaves of transgenic plants of DJ background overexpressing *OsOxO2* and with significantly increased OxO activity. Moreover, in these overexpression transgenic plants, there was no significant auxin deficiency phenotype which was present in *lc*4, *IAAGLU*OE/DJ plants, while there only slightly enlarged leaf angle and decreased plant height. Interestingly, in *Drosophila melanogaster*, *Acsx1L* (CG6300) and *Acsx1R* (CG11659) are tandem duplicates of a putative acyl-CoA synthetase gene, and *Acsx1L* expression is substantially reduced upon deleting the right-hand duplicated block *Acsx1R* (Loehlin et al. 2022). Moreover, tandem duplication often results in more than double the gene activity (Loehlin and Carrolla 2016). In view of the relative position of *OsIAAGLU*, *OsOxO1*, *OsOxO2* and *OsOxO3* on chromosome 3, we would speculate that *OsOxO2* might affect the levels of *OsOxO1* and *OsIAAGLU* transcripts in a position-dependent manner. Mutation in *OsOxO2* might influence the expression of *OsOxO1* and *OsOxO3*, and the change in expression of *OsOxO1* might then influence the expression of *OsIAAGLU*. Because gain or loss of the accessible flanking chromatin regions (ACRs) and mutation of cis-regulatory elements (CREs) within ACRs can change the balance of the expression level and/or tissue specificity of the duplicated genes (Fang et al. 2023). Moreover, the genes that are clustered closely in the genome often tend to be co-regulated and exhibit correlated expression, while neighboring genes might also show correlated expression change (Ghanbarian and Hurst 2015). It has been suggested that the expression of one gene could directly impact on the expression of its neighboring genes in the immediate vicinity and this effect is stronger for tandem duplicates which are often co-regulated by shared genomic elements (Lan and Pritchard 2016). However, the underlying mechanism of chromosome position-dependent gene expression changes is still elusive and remains to be explored further.

## Supplementary Information

Below is the link to the electronic supplementary material.

Supplementary Material 1. Figure S1 PCR product sequence amplified from *lc*4 mutant using leaf genomic DNA as template and LB (T-DNA-specific) and RP (*OsOxO2* specific) as primers. The blue-highlighted bases represent the 270-nucleotide region upstream of the *OsOxO2* start codon (ATG). Figure S2 T-DNA insertion in 270 nucleotides upstream from the *OsOxO2* start codon (ATG) resulted in enhanced OxO activity and OsOxO2 abundance. (A) OxO isoforms in rice leaves were separated by 7.5% CN-PAGE and visualized by in-gel OxO activity staining. Leaf extracts were analyzed from the following plants: wild-type Zhonghua 11 (ZH11) and Dongjin (DJ), transgenic plants overexpressing (OE) *OsOxO1*, *OsOxO2*, *OsOxO3*, or *OsOxO4* in the ZH11 background, and *OsOxO2*OE plants in the DJ background, the *lc*4 mutant and hybrid plants generated by crossing *OsOxO1*OE and *OsOxO2*OE (ZH11 background) lines. (B) immunoblot analysis of proteins separated by 12.5% SDS-PAGE. Leaf extracts from the same genotypes as in (A) were probed with an anti-OsOxO4-His antibody. Figure S3 Characterization of the *lc*4 T-DNA insertion mutant at the 4-leaf stage and flag leaf lamina joints at the booting time. (A) phenotype of the 4-leaf stage representative rice variety Dongjin (DJ) wild-type and *lc*4 plants. (B) and (C) quantification of leaf angle and plant height of plants depicted in (A). (D) adaxial/abaxial phenotype of flag leaf lamina joints at the booting time. (E) quantification of adaxial/abaxial distance depicted in (D). Data are presented as means ± SD. Asterisks indicate statistically significant differences (Student’s *t*-test, *P* < 0.0001). Figure S4 Co-segregation analysis of the phenotype of the progeny from heterozygous PFG_3A-02040.L mutant 3A9-2: (A) phenotypes of progeny of heterozygous PFG_3A-02040.L mutant 3A9: 3A9-3 was homozygous, and its OxO activity in the leaves was higher than 3A9-2 which was heterozygous. There was no T-DNA insertion in 3A9-1 and no OxO activity was detected in its leaves. (B) primers sets (LP + RP and LB + RP) were used for genotyping part of progeny lines of the heterozygous PFG_3A-02040.L mutant 3A9-2 by PCR amplification using genomic DNA as template. (C) OxO activity in leaves and leaf angle (D) of progeny lines of the heterozygous PFG_3A-02040.L mutant 3A9-2 at the 4-leaf-stag. Figure S5 Mutation of *OsIAAGLU* or *OsOxO2* in the *lc*4 background can restore its leaf angle phenotype similar to 3-leaf-stage wild-type (DJ) seedlings. (A) phenotypes of whole plants and leaf angle of representative seedlings. (B) and (C) quantification of leaf angle and plant height of seedlings depicted in (A). Figure S6 Identification of *iaaglu* and *oxo2* mutant plants in *lc*4 background. (A) the sgRNA target region of *oxo2*/*lc*4 and *iaaglu*/*lc*4. The numbers on the bases represent its position in *OsIAAGLU* or *OsOxO2* CDS, the inserted base is marked with red. (B) primer sets (LP + RP and LB + RP) were used for genotyping part of *oxo2*/*lc4* and *iaaglu*/*lc*4 by PCR amplification using leaf genomic DNA as template. Table S1 Primer sequences used for PCR and vector construction.

## Data Availability

No datasets were generated or analysed during the current study.
