# Supplementary material for: Activation of OsOxO2 by T-DNA Insertion Affects Plant Height and Leaf Angle in Rice
Source: Rice (N Y). 2026 Jan 19;19:12. doi: 10.1186/s12284-025-00879-0 (PMC12894459; doi:10.1186/s12284-025-00879-0)
Supplement: Supplementary file 1 — Supplementary Material 1. Figure S1 PCR product sequence amplified from lc4 mutant using leaf genomic DNA as template and LB (T-DNA-specific) and RP (OsOxO2 specific) as primers. The blue-highlighted bases represent the 270-nucleotide region upstream of the OsOxO2 start codon (ATG). Figure S2 T-DNA insertion in 270 nucleotides upstream from the OsOxO2 start codon (ATG) resulted in enhanced OxO activity and OsOxO2 abundance. (A) OxO isoforms in rice leaves were separated by 7.5% CN-PAGE and visualized by in-gel OxO activity staining. Leaf extracts were analyzed from the following plants: wild-type Zhonghua 11 (ZH11) and Dongjin (DJ), transgenic plants overexpressing (OE) OsOxO1, OsOxO2, OsOxO3, or OsOxO4 in the ZH11 background, and OsOxO2OE plants in the DJ background, the lc4 mutant and hybrid plants generated by crossing OsOxO1OE and OsOxO2OE (ZH11 background) lines. (B) immunoblot analysis of proteins separated by 12.5% SDS-PAGE. Leaf extracts from the same genotypes as in (A) were probed with an anti-OsOxO4-His antibody. Figure S3 Characterization of the lc4 T-DNA insertion mutant at the 4-leaf stage and flag leaf lamina joints at the booting time. (A) phenotype of the 4-leaf stage representative rice variety Dongjin (DJ) wild-type and lc4 plants. (B) and (C) quantification of leaf angle and plant height of plants depicted in (A). (D) adaxial/abaxial phenotype of flag leaf lamina joints at the booting time. (E) quantification of adaxial/abaxial distance depicted in (D). Data are presented as means ± SD. Asterisks indicate statistically significant differences (Student’s t-test, P < 0.0001). Figure S4 Co-segregation analysis of the phenotype of the progeny from heterozygous PFG_3A-02040.L mutant 3A9-2: (A) phenotypes of progeny of heterozygous PFG_3A-02040.L mutant 3A9: 3A9-3 was homozygous, and its OxO activity in the leaves was higher than 3A9-2 which was heterozygous. There was no T-DNA insertion in 3A9-1 and no OxO activity was detected in its leaves. (B) prim [file 12284_2025_879_MOESM1_ESM.doc]

**
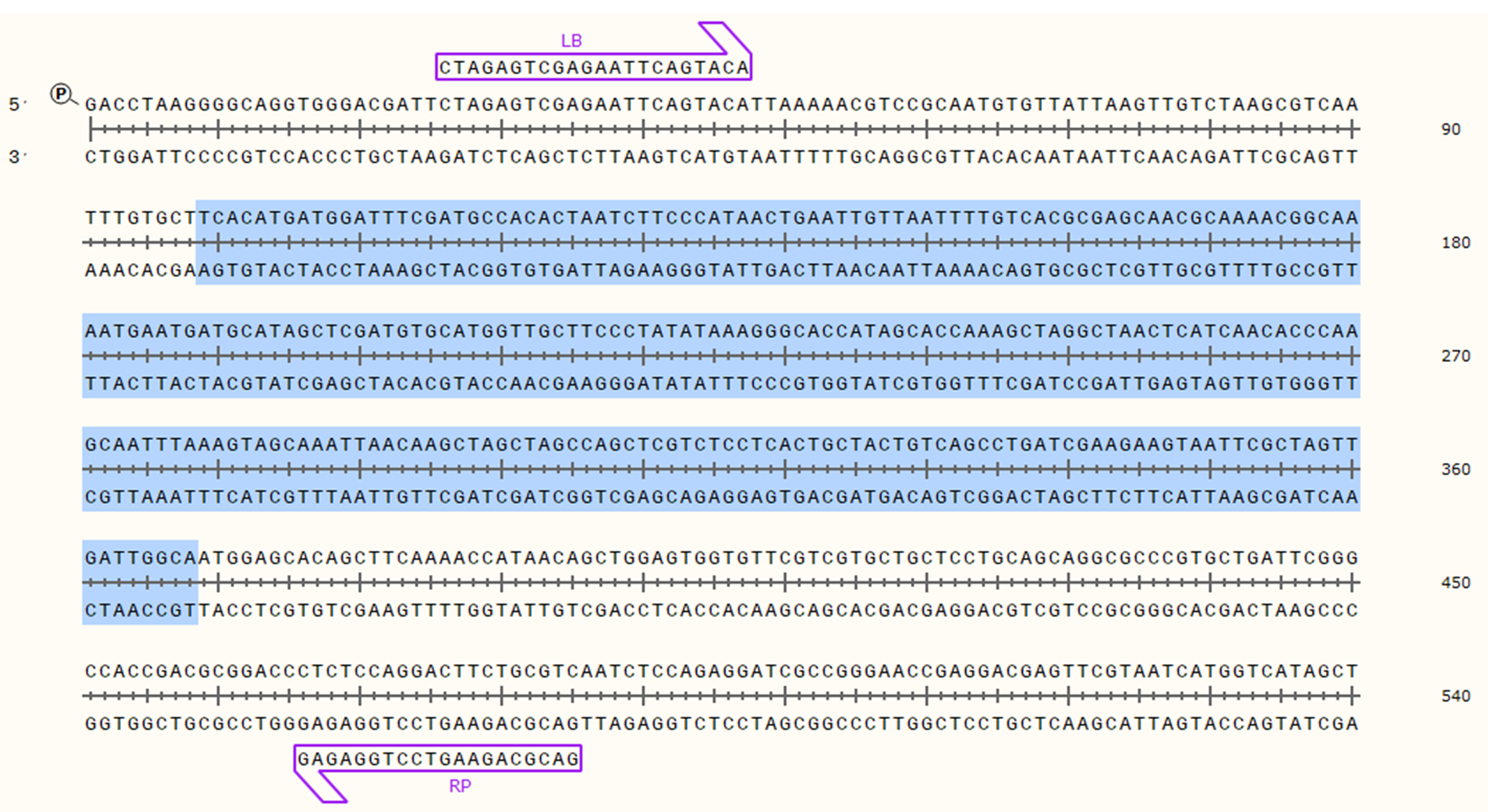
**

**Figure S1** PCR product sequence amplified from *lc4* mutant using leaf genomic DNA as template and LB (T-DNA-specific) and RP (*OsOxO2* specific) as primers. The blue-highlighted bases represent the 270-nucleotide region upstream of the *OsOxO2* start codon (ATG).


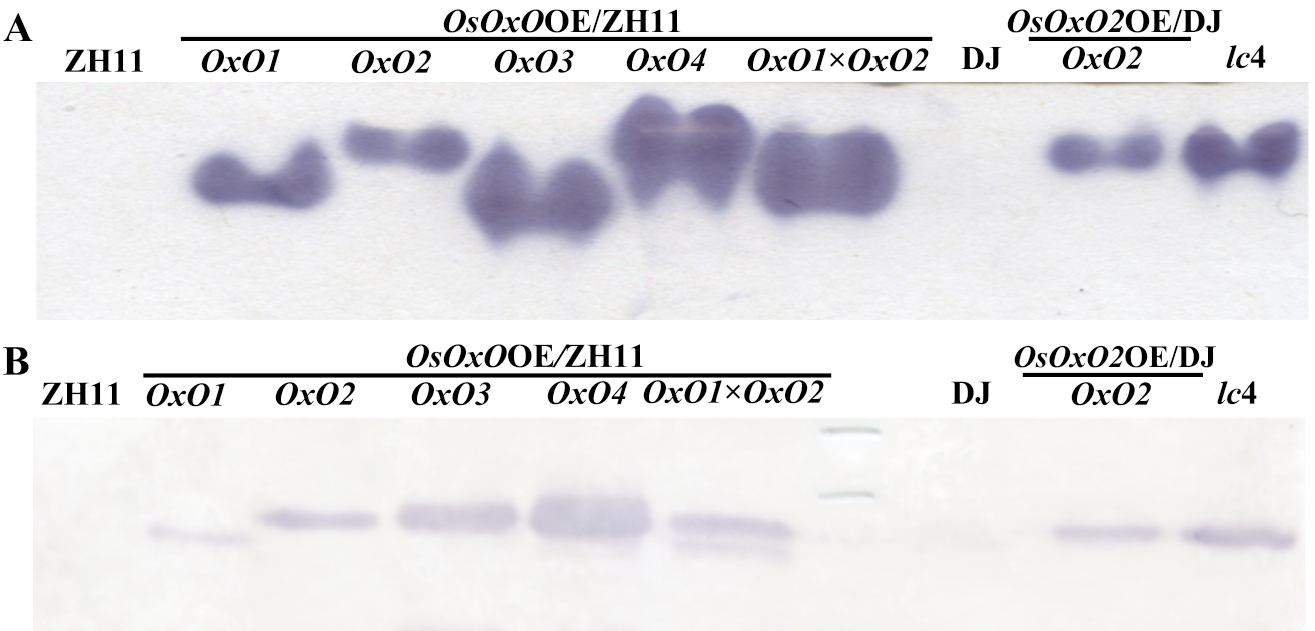


**Figure S2** T-DNA insertion in 270 nucleotides upstream from the *OsOxO2* start codon (ATG) resulted in enhanced OxO activity and OsOxO2 abundance. (A) OxO isoforms in rice leaves were separated by 7.5% CN-PAGE and visualized by in-gel OxO activity staining. Leaf extracts were analyzed from the following plants: wild-type Zhonghua 11 (ZH11) and Dongjin (DJ), transgenic plants overexpressing (OE) *OsOxO1*, *OsOxO2*, *OsOxO3*, or *OsOxO4* in the ZH11 background, and *OsOxO2*OE plants in the DJ background, the *lc*4 mutant and hybrid plants generated by crossing *OsOxO1*OE and *OsOxO2*OE (ZH11 background) lines. (B) immunoblot analysis of proteins separated by 12.5% SDS-PAGE. Leaf extracts from the same genotypes as in (A) were probed with an anti-OsOxO4-His antibody.


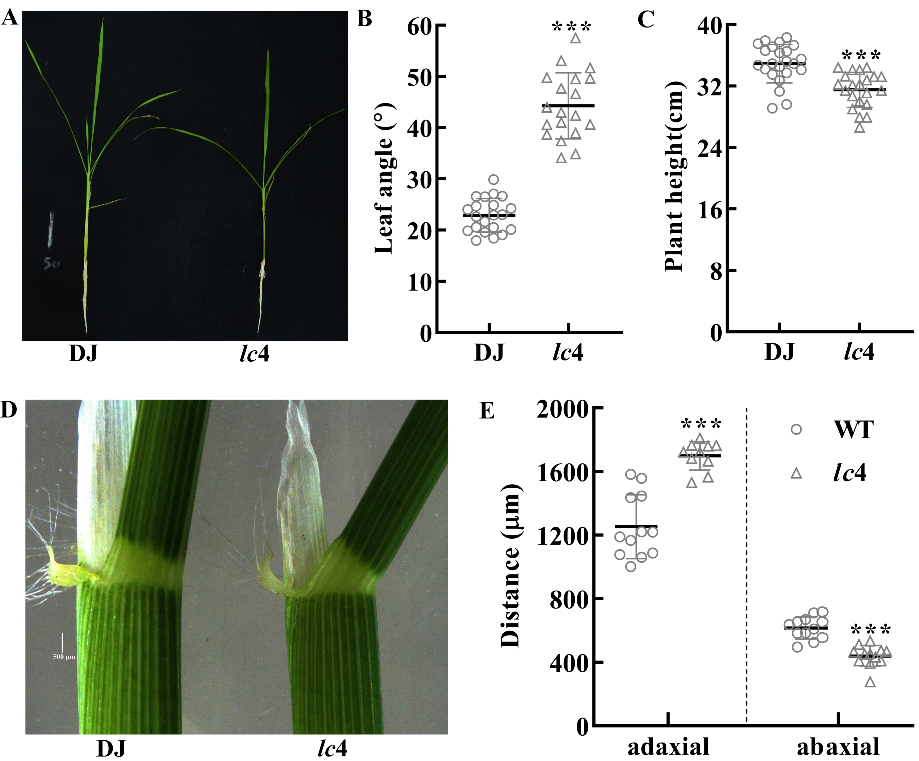


**Figure S3** Characterization of the *lc*4 T-DNA insertion mutant at the 4-leaf stage and flag leaf lamina joints at the booting time. (A) phenotype of the 4-leaf stage representative rice variety Dongjin (DJ) wild-type and *lc*4 plants. (B) and (C) quantification of leaf angle and plant height of plants depicted in (A). (D) adaxial/abaxial phenotype of flag leaf lamina joints at the booting time. (E) quantification of adaxial/abaxial distance depicted in (D). Data are presented as means ± SD. Asterisks indicate statistically significant differences (Student’s *t*-test, *P*<0.0001).


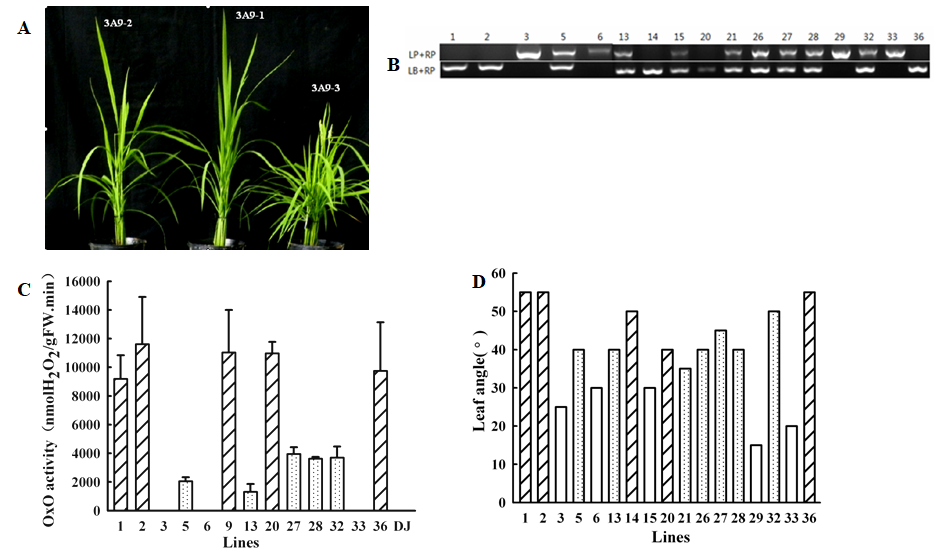


**Figure S4** Co-segregation analysis of the phenotype of the progeny from heterozygous PFG_3A-02040.L mutant 3A9-2: (A) phenotypes of progeny of heterozygous PFG_3A-02040.L mutant 3A9: 3A9-3 was homozygous, and its OxO activity in the leaves was higher than 3A9-2 which was heterozygous. There was no T-DNA insertion in 3A9-1 and no OxO activity was detected in its leaves. (B) primers sets (LP+RP and LB+RP) were used for genotyping part of progeny lines of the heterozygous PFG_3A-02040.L mutant 3A9-2 by PCR amplification using genomic DNA as template. (C) OxO activity in leaves and leaf angle (D) of progeny lines of the heterozygous PFG_3A-02040.L mutant 3A9-2 at the 4-leaf-stag.


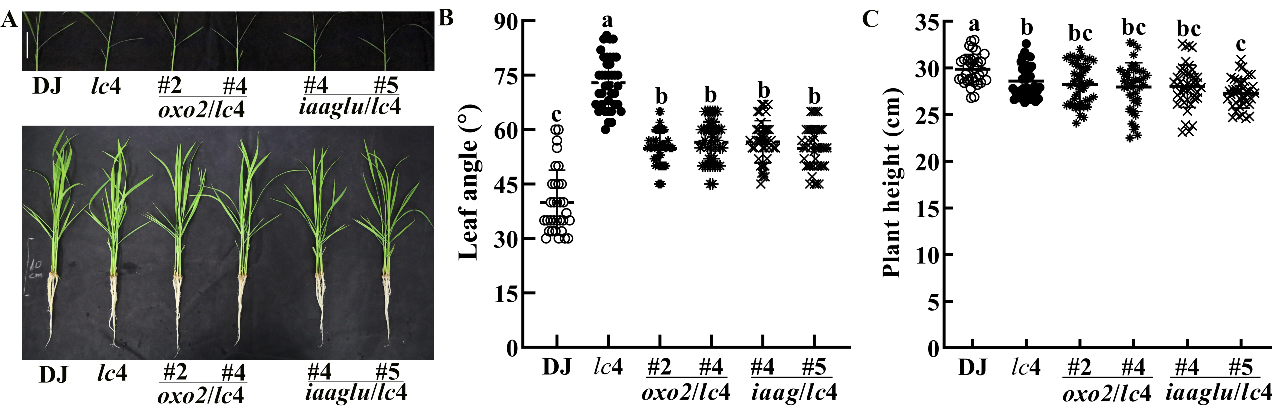


**Figure S5** Mutation of *OsIAAGLU* or *OsOxO2* in the *lc*4 background can restore its leaf angle phenotype similar to 3-leaf-stage wild-type (DJ) seedlings. (A) phenotypes of whole plants and leaf angle of representative seedlings. (B) and (C) quantification of leaf angle and plant height of seedlings depicted in (A).


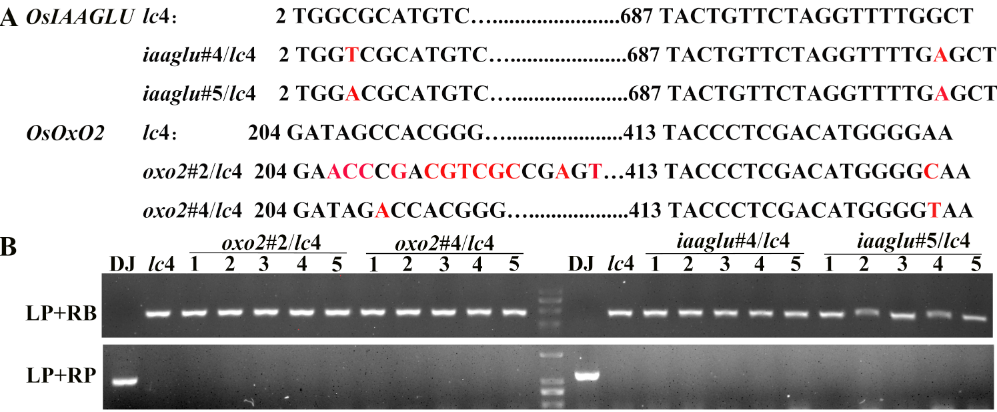


**Figure S6** Identification of *iaaglu* and *oxo2* mutant plants in *lc*4 background. (A) the sgRNA target region of *oxo2*/*lc*4 and *iaaglu*/*lc*4. The numbers on the bases represent its position in *OsIAAGLU* or *OsOxO2* CDS, the inserted base is marked with red. (B) primer sets (LP+RP and LB+RP) were used for genotyping part of *oxo2*/*lc*4 and *iaaglu*/*lc*4 by PCR amplification using leaf genomic DNA as template.

Table S1 Primer sequences used for PCR and vector construction

| Name of primers | Sequences(5'-3) |
| --- | --- |
| LP | CCCTCCATTCCAAATTGATC |
| RP | GACGCAGAAGTCCTGGAGAG |
| LB | CTAGAGTCGAGAATTCAGTACA |
| q*IAAGLU-*L | TGGACGAGTTCGTGGAGTTC |
| q*IAAGLU-*R | TCACATCTCCGACGCTGCAG |
| q*ACTIN*-L | CTTCATAGGAATGGAAGCTGCGGGTA |
| q*ACTIN*-R | CGACCACCTTGATCTTCATGCTGCTA |
| *OsOxO1*-S | CCAACGTCACGGAGCTCGACGTC |
| *OsOxO1*-A | CGGCAAGACGGAGGCCACCATGG |
| *OsOxO2*-S | CCAACGTCACGGAGCTCGACGTC |
| *OsOxO2*-A | CGGCAAGACGGAGGCCACCATGG |
| *OsOxO4*-S | CCAACGTCACGGAGCTCGACGTC |
| *OsOxO4-*A | CGGCAAGACGGAGGCCACCATGG |
| *OsOxO2*OE-L | GCGCAAGCTTATGGAGCACAGCTTCAA |
| *OsOxO2*OE-R | AGCGGATCCTTAGTACCCACCGGTGAAC |
| Bar-65-L | GCACCATCGTCAACCACTAC |
| Bar-491-R | CAGAAACCCACGTCATGC |
| Cas9-LP | CACTAAGGCTCCTCTTTCTG |
| Cas9-RP | TGACGTACTTGACCTTGGTG |
